# Modification of chitooligosaccharide with phenolic acids for extended cherry tomato preservation and quality retention

**DOI:** 10.1016/j.isci.2026.114797

**Published:** 2026-01-24

**Authors:** Ruyi Zhang, Hui Chen, Ruohui Li, Shuyan Lin, Nan Wang

**Affiliations:** 1 Department of Biological Environment, Jiyang College of Zhejiang A&F University, Zhuji, Zhejiang 311800, P.R. China; 2 School of Forestry & Biotechnology, Zhejiang A&F University, Hangzhou, Zhejiang 311300, P.R. China

**Keywords:** Natural sciences, Plant biochemistry, Bioactive plant product, Agricultural plant products

## Abstract

Coating materials extend fruit shelf life by forming protective barriers against microbial growth and preserving freshness. Herein, three chitooligosaccharide (COS) derivatives were synthesized via grafting protocatechuic acid, caffeic acid, and gallic acid onto COS, with their structures characterized. Fourier transform infrared spectroscopy confirmed successful grafting via amide linkages. ^1^H NMR analysis revealed new absorption peaks between 6.5 and 7.5 ppm, attributable to the grafted phenolic acids. Compared to ungrafted COS, the derivatives exhibited enhanced antioxidant activity and superior bacteriostatic activity. Spraying 2 mg L^−1^ derivatives on cherry tomatoes significantly reduced decay (33.7%–44.7%), browning (14.3%–23.6%), and the accumulation of reducing sugars (41.9%–49.6%) and soluble solids (13.3%–22.3%) during storage. These derivatives alleviated oxidative stress by enhancing catalase activity and reducing malondialdehyde levels, while inhibiting polyphenol oxidase to slow phenolic degradation and delay senescence. This grafted COS derivative coating offers enhanced biocompatibility for cherry tomato preservation systems.

## Highlights

• Grafted COS derivatives enhance antioxidant and antibacterial activities
• Derivative coatings significantly reduce cherry tomato decay and browning
• Derivatives offer enhanced biocompatibility for cherry tomato preservation systems

## Graphical abstract

**Figure:**
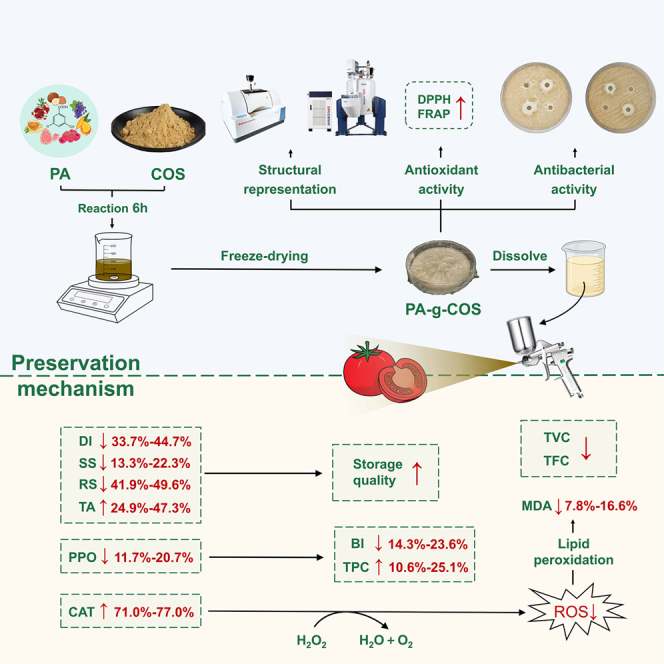

## Introduction

The tomato (*Solanum lycopersicum*) is one of the world’s most important vegetables, valued for its bright color, unique flavor, and abundant health-beneficial bioactive compounds. The bioactive compounds of tomatoes encompass carotenoids, ascorbic acid, and phenolic compounds. Carotenoids act as vitamin A precursors, supporting visual function, somatic growth, and immune regulation.^1^^,^^2^ Meanwhile, ascorbic acid and phenolic compounds exert significant antioxidant activity through free radical scavenging, reducing the risk of chronic diseases, including cardiovascular disorders and malignancies.^3^ Therefore, effective postharvest preservation of tomatoes is crucial for ensuring commercial viability for producers and retaining maximal nutritional value for global consumers.

As a typical climacteric fruit, tomatoes face considerable challenges in postharvest management. Following harvest, fresh tomatoes engage in respiration, undergoing a series of physiological metabolic activities during storage, resulting in a loss of nutritional value and commercial worth due to a decline in overall quality.^4^ Meanwhile, high-humidity environments facilitate colonization by pathogenic microorganisms (e.g., *Botrytis cinerea*), resulting in 40%–50% postharvest losses.^5^ Traditional petroleum-based plastics are widely applied in food packaging and storage, benefiting from their tunable mechanical properties and excellent gas barrier characteristics.^6^ However, with the development of the economy, public demands and expectations for green, safe food are becoming more and more specific. The poor degradability and microplastic residue release of traditional petroleum-based plastics conflict with the contemporary principles of sustainable development and the global pursuit of carbon emission reduction. Therefore, developing biodegradable packaging materials to substitute traditional petroleum-based plastics would alleviate environmental pressure and address public worries about the microplastic residues of food packaging.^7^ Current biodegradable packaging materials (films and coatings) primarily encompass polysaccharides and proteins, e.g., cellulose, chitosan (CS), pectin, alginate, gelatin, whey protein, and zein. CS is a product derived from the deacetylation of chitin. Chitin is widely present in nature and is the second most abundant natural polymer after cellulose, with an estimated annual biosynthesis of one billion tonnes. As a result, CS is regarded as a highly promising renewable resource.^8^^,^^9^^,^^10^ CS exhibits broad-spectrum antibacterial ability, excellent biocompatibility, and a promising biopolymer molecule to replace plastic food packaging.^11^ However, the poor solubility of CS in neutral pH and its high viscosity limit its further application in postharvest fruit preservation.

**Figure 1:**
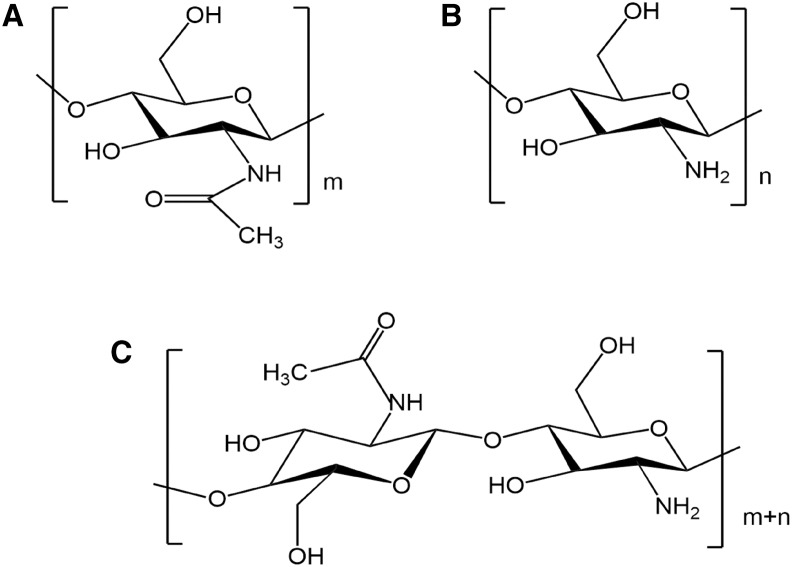
Structure of COS (A) N-acetylated COS. (B) N-deacetylated COS. (C) Partially N-deacetylated COS.

In recent years, chitooligosaccharide (COS), a natural biostimulator, has been widely applied in the postharvest storage of horticultural products. As a low molecular weight compound obtained by chemical, enzymatic, or radiation degradation of CS (Figure 1), COS has a degree of polymerization (DP) < 20 and average molecular weight (Mw) < 3.9 kDa. Each D-glucosamine unit of COS contains a free amino group, and this structural feature endows COS with excellent film-forming ability, water solubility, and biological activities (e.g., degradability, antibacterial, and antioxidant properties).^12^ Consequently, COS is widely applied in medicine, food, agriculture, and wastewater treatment. As a natural cationic polysaccharide, COS inhibit fungal growth by forming polyelectrolyte complexes with negatively charged groups on cell surfaces and induce postharvest defense responses in fruits to reduce pathogen infection.^13^ However, the relatively weak antioxidant activity of COS limits its application in food preservation.^14^ The active C-2 amino group, C-3 primary hydroxyl group, and C-6 secondary hydroxyl group in COS can serve as modification sites for chemical modification, thereby enhancing the antioxidant and other biological activities of COS.^15^ COS graft copolymerization reactions are an effective method to endow COS with the desired physicochemical and biological properties.^16^ Considering the safety of copolymers, grafting phenolic acids onto COS to form graft copolymers is a common strategy to improve the antibacterial and antioxidant properties of COS.^17^

Phenolic acids (PAs), a class of important plant-derived secondary metabolites, exhibit a variety of physiological activities, including antioxidant, antibacterial, anti-inflammatory, antitumor, and free radical-scavenging activities.^18^^,^^19^ The presence of phenolic hydroxyl groups endows them with excellent antioxidant capacity, as these groups can donate hydrogen atoms to terminate oxidation chains.^20^ However, the poor stability, low biological permeability, and low water solubility of phenolic acids lead to low bioavailability and limited utilization efficiency.^21^^,^^22^ In this regard, taking advantage of the biocompatibility and water solubility of COS, chemical modification with phenolic acids can compensate for the insufficient antioxidant activity of COS.^23^ However, COS grafted with phenolic acids remains significantly unexplored for cherry tomato preservation, despite their demonstrated potential in other food applications due to their antioxidant activity.^24^

The objective of this study is to screen phenolic acid-grafted chitooligosaccharide (PA-g-COS) derivatives with ideal performance, and to investigate the effects of different derivatives on the preservation of cherry tomatoes. To achieve this objective, three phenolic acids, including protocatechuic acid (PCA), caffeic acid (CFA), and gallic acid (GLA), were selected according to their type and the number of hydroxyl groups. The derivatives prepared by the radical-mediated grafting method were characterized by Ultraviolet-visible spectrum (UV-Vis), Fourier transform infrared (FTIR), and Proton nuclear magnetic resonance (^1^H NMR) analyses. Subsequently, the performance of different phenolic acid-grafted COS derivatives was compared through *in vitro* tests of antioxidant, antibacterial, and cytotoxicity properties. Furthermore, the preservation mechanisms of these derivatives in cherry tomatoes were evaluated. Overall, this study systematically investigates the preservation performance of phenolic acid-grafted COS derivatives on cherry tomatoes, providing a new technical option aligned with sustainable development concepts for the postharvest preservation of small berry fruits.

## Results and discussion

### Structural characterization of different phenolic acid-grafted chitooligosaccharide derivatives

**Figure 2:**
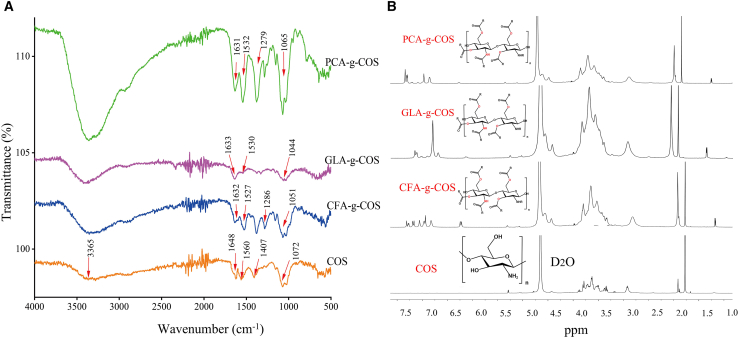
Spectroscopic characterization of different PA-g-COS derivatives (A) FTIR spectra of different PA-g-COS derivatives. (B) ^1^H NMR spectra of different PA-g-COS derivatives. *p* value notification: ∗*p* < 0.05, ∗∗*p* < 0.01 and ∗∗∗*p* < 0.001.

The chemical structures of phenolic acid-grafted chitooligosaccharide (PA-g-COS) derivatives were characterized by FTIR spectroscopy and compared with those of COS (Figure 2A). In the spectrum of COS, the absorption band at 3365 cm^−1^ corresponds to the stretching vibration of the -OH group. The characteristic peak at 1560 cm^−1^ is due to the N-H stretching vibration of the amide II bond, while the peak at 1648 cm^−1^ arises from the C=O stretching vibration of the amide I bond.^25^ The peak at 1407 cm^−1^ is attributed to the N-H absorption of the amide III bond. The absorption at 1072 cm^−1^ is the stretching vibration of the glucoside C-*O*-C bond between the chitosan monomers and glucose.^26^ Compared to ungrafted COS, grafted derivatives exhibit a reduced intensity at 1648 cm^−1^ (amide I) and a new characteristic peak at ∼1630 cm^−1^, which is ascribed to the overlapping vibrations of the phenolic acid benzene ring and C=O stretching.^27^ The attenuated absorbance at 1648 cm^−1^ (amide I) and enhanced peak at ∼1630 cm^−1^ collectively indicate the formation of secondary amides via covalent bonding conjugation between the amino groups of COS and the carboxyl groups of phenolic acids.^28^ Concurrently, the decreased intensity of the amide II peak at 1560 cm^−1^ and the emergence of a new peak at 1527-1532 cm^−1^ confirm that covalent modification occurred at the amino (-NH_2_) sites of COS. These spectral changes verify successful grafting of PAs onto COS via amide linkages.^28^ ^1^H NMR spectroscopy further confirms the grafting reaction (Figure 2B). Comparison of the ^1^H NMR spectra of COS and PA-g-COS derivatives revealed that all PA-g-COS samples exhibit one or more sets of aromatic proton signals at 6.5–7.5 ppm, which originate from the benzene rings of phenolic acid molecules.^27^ This result suggests the successful grafting of phenolic acids onto the COS molecular framework.

This structural modification significantly enhances the stability of the derivatives. When phenolic acid molecules are incorporated into the COS framework, they form hydrogen bonds with COS chains.^16^ These intermolecular interactions strengthen the thermal stability of the derivatives, which exhibits a positive correlation with the degree of substitution.^17^ The influence of pH on the stability of PA-g-COS derivatives was investigated during storage (Figure S1). Notably, compared to ungrafted COS, PA-g-COS derivatives exhibit superior stability across different pH conditions.

### Yields and the degree of substitution of different phenolic acid-grafted chitooligosaccharide derivatives

After obtaining the target PA-g-COS derivatives, their yields were calculated, with the results shown in Table 1. Additionally, the degree of substitution (DS) of each derivative was determined via ^1^H NMR spectroscopy: the [H1] signal of COS was selected as the integral standard peak, and DS values were calculated based on the ratio of the characteristic peak area of the grafted phenolic acid to that of the [H1] signal. These DS results were also shown in Table 1.

**Table 1:** The yields and DS of PA-g-COS derivatives

Compounds	Yields (%)	DS (%)
CFA-g-COS	24.10%	77.61%
PCA-g-COS	28.17%	68.80%
GLA-g-COS	32.65%	27.95%

### Antioxidant activity of different phenolic acid-grafted chitooligosaccharide derivatives

**Figure 3:**
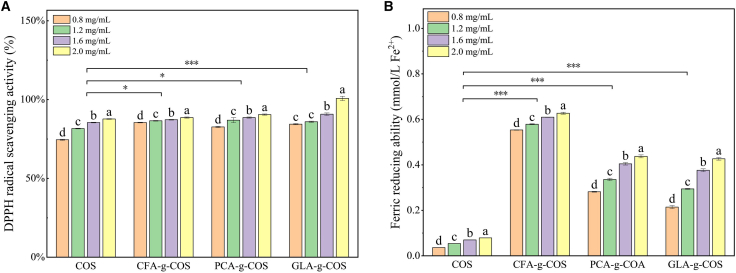
Antioxidant properties of different PA-g-COS derivatives (A) DPPH free radical scavenging ability of different PA-g-COS derivatives. (Data are presented as mean ± SD, *n* = 3 independent experiments, one-way ANOVA followed by Tukey’s test). (B) Ferric reducing antioxidant power (FRAP) of different PA-g-COS derivatives. (Data are presented as mean ± SD, *n* = 3 independent experiments, one-way ANOVA followed by Tukey’s test). Different lowercase letters indicate significant differences in different concentrations between the same treatment groups (*p* < 0.05). *p* value notification: ∗*p* < 0.05, ∗∗*p* < 0.01 and ∗∗∗*p* < 0.001.

As shown in Figure 3A, PA-g-COS derivatives exhibited concentration-dependent DPPH radical scavenging activity, with their efficacy increasing as the concentration rose. At 2 mg mL^−1^, the GLA-g-COS, PCA-g-COS, and CFA-g-COS treatments achieved maximum scavenging rates of 99.95%, 90.50%, and 88.60%, respectively. These values represent increases of 14.8%, 12.8%, and 13.2% compared to ungrafted COS (87.02%) treatment. In the concentration range of 0–2.0 mg mL^−1^, the DPPH radical scavenging capacity followed the order: GLA-g-COS > PCA-g-COS > CFA-g-COS.

The ferric reducing antioxidant power (FRAP) assay results (Figure 3B) further confirmed the potent antioxidant capacity of PA-g-COS derivatives. At a concentration of 2 mg mL^−1^, CFA-g-COS exhibited the strongest ferric ion-reducing ability (0.61 mmol L^−1^), which was approximately 8-fold higher than that of ungrafted COS (0.08 mmol L^−1^). In the concentration range of 0–2.0 mg mL^−1^, the ferric reducing capacity of the derivatives followed the order: CFA-g-COS > PCA-g-COS > GLA-g-COS.

The concentration-dependent DPPH radical scavenging activity of PA-g-COS derivatives is consistent with the finding of Wang et al.,^29^ confirming a distinct concentration-dependent pattern in their antioxidant activity. As shown in Figure 3A, phenolic acid modification significantly enhances the radical scavenging capacity of COS, primarily attributed to the hydrogen-donating ability of phenolic hydroxyl groups. These groups react with DPPH radicals to form stable semiquinone structures, thereby interrupting the oxidative chain reaction.^23^ This observation aligns with Huerta-Madronal et al.,^30^ who highlighted the decisive role of phenolic acid structures in regulating the antioxidant activity of COS derivatives. Previous studies^31^^,^^32^ have demonstrated that the degree and position of hydroxylation in phenolic compounds significantly influence antioxidant potential. In the present study, although the three phenolic acids possess a similar degree of hydroxylation, their specific hydroxylation positions emerged as critical factors regulating the antioxidant capacity of PA-g-COS derivatives. Notably, the CFA-g-COS derivative exhibited relatively weaker antioxidant activity compared to the other two derivatives, which can be attributed to its lower DPPH radical scavenging efficiency.

The FRAP assay, which evaluates ferric ion-reducing ability, revealed an antioxidant profile characterized by electron-donating and oxidant-neutralizing abilities. For ungrafted COS, FRAP values increased with concentration (0.8–2.0 mg mL^−1^), consistent with previously reported concentration-dependent antioxidant behavior.^33^ This indicates that higher COS concentrations provide more electron-donating sites (e.g., hydroxyl groups) for reduction reactions. Compared to ungrafted COS, phenolic acid-grafted COS derivatives (CFA-g-COS, PCA-g-COS, GLA-g-COS) exhibited significantly enhanced FRAP values, confirming that phenolic acid modification boosts the reducing power of COS. This enhancement may involve two synergistic mechanisms: (1) the high positive charge density of grafted derivatives promotes electron transfer efficiency^34^; (2) the benzene ring structure of phenolic acids inhibits lipid peroxidation via free radical chain termination.^35^ Among the three derivatives, CFA-g-COS exhibited the highest FRAP levels, indicating stronger electron-donating ability. In contrast, although GLA-g-COS showed concentration-dependent increases in FRAP values, its performance was lower than the other two derivatives at equivalent concentrations. This observation may be attributed to the hydroxylation position and steric hindrance of GLA, which limit Fe^3+^ access to its phenolic hydroxyl groups and thereby reduce electron transfer efficiency.

### Antibacterial activity of different phenolic acid-grafted chitooligosaccharide derivatives

**Figure 4:**
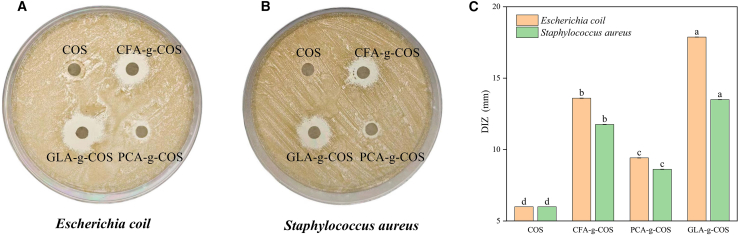
Antibacterial activity of different PA-g-COS derivatives (A and B) *Escherichia coli* and *Staphylococcus aureus* antibacterial images. (C) Diameters of the inhibition zones (DIZs) of different PA-g-COS derivatives. (Data are presented as mean ± SD, *n* = 3 independent experiments). Different lowercase letters indicated significant differences between different treatment groups of the same strain (*p* < 0.05).

The antibacterial activities of COS and PA-g-COS derivatives against Gram-negative and Gram-positive bacteria are presented in Table S1, with results expressed as minimum inhibitory concentration (MIC). COS exhibited higher MIC values than all PA-g-COS derivatives, indicating that the derivatives had stronger bacterial inhibitory activity than ungrafted COS. The diameter of the inhibition zone (DIZ) further confirmed that phenolic acid-grafted COS derivatives significantly enhanced the antibacterial activity of COS at the same concentration (Figures 4A and 4B). Similar findings were reported when COS was conjugated with ferulic acid, which resulted in enhanced antibacterial activity.^17^ One possible reason is that the introduction of phenolic acids improves bacteriostasis efficacy, as phenolic acids themself possess inherent antibacterial properties. Among all the derivatives, GLA-g-COS showed the lowest MIC value, and the largest DIZ (Figure 4C), which indicates superior antibacterial activity against all tested bacterial strains.

### Biocompatibility of different phenolic acid-grafted chitooligosaccharide derivatives

**Figure 5:**
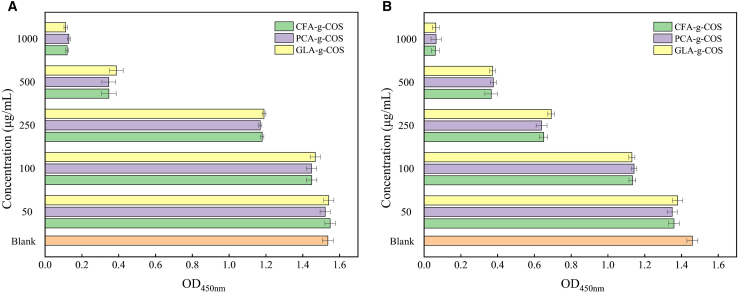
The cytotoxicity of different PA-g-COS derivatives using the CCK8 assay (A) GSE-1 cells. (Data are presented as mean ± SD, *n* = 6 independent experiments). (B) NCM-460 cells. (Data are presented as mean ± SD, *n* = 6 independent experiments).

To evaluate the biocompatibility of phenolic acid-grafted COS derivatives, *in vitro* cytotoxicity assays were performed using human gastric mucosal epithelial cells (GES-1) and normal human colonic epithelial cells (NCM-460). As shown in Figure 5, both cell lines displayed concentration-dependent viability changes in response to phenolic acid-g-COS treatment. For GES-1 cells, treatment with 50 μg mL^−1^ PA-g-COS derivatives showed a minimal impact on cell viability, with the cell survival rate comparable to the control group (Figure 5A). At a concentration of 100 μg mL^−1^, cell viability remained at 94%–96% of the control level. Even when the concentration was increased to 250 μg mL^−1^, the cell survival rate was still maintained at 74%–79% of the control level. Only at the maximum tested concentration (1000 μg mL^−1^) was cell viability substantially reduced to 7%–8% of the control level. NCM-460 cells exhibited poor tolerance to phenolic acid-grafted COS derivatives (Figure 5B). Elevating the concentration to 250 μg mL^−1^ led to a significant reduction in cell survival rate to 48%–50%, relative to the control. At the maximum tested concentration (1000 μg mL^−1^), cell viability was severely impaired, with only 2%–3% of cells remaining viable relative to the untreated control group. To address safety concerns, a comprehensive assessment of phenolic acid-grafted COS derivatives exposure levels and potential risks was conducted. In the coating application, cherry tomatoes were treated with a 2 mg mL^−1^ phenolic acid-grafted COS derivative solution, with approximately 0.5 mL adhering per 14 g of cherry tomatoes (surface area ∼20–30 cm^2^), resulting in an estimated surface concentration of 35–50 μg cm^−2^. Given typical cherry tomato consumption patterns where the whole fruit is eaten directly, the potential consumer exposure to the derivative coating was evaluated. Even considering potential derivative migration to the edible fruit tissue, the actual exposure levels would be substantially lower than the concentrations evaluated in the cytotoxicity assays (up to 100 μg mL^−1^), which demonstrated no significant toxicity.

To further investigate the fate of phenolic acid-grafted COS derivatives, residual derivatives on cherry tomato surfaces were analyzed after 6 days of storage. Thirty treated cherry tomatoes were selected, and their surfaces were thoroughly rinsed with deionized water three times. The combined rinsates were lyophilized, and the results confirmed that approximately 80% of the applied derivative remained recoverable from the cherry tomato surface. While the exact fate of the remaining 20% could not be definitively determined due to the complex composition of cherry tomato peels and the physiological metabolic activities of postharvest fruits, two likely scenarios exist: degradation into COS and phenolic acid monomers or migration into the fruit pulp tissue. Importantly, both scenarios present minimal safety concerns. The phenolic acid-grafted COS derivatives are synthesized from food-grade raw materials, natural COS, and plant-derived phenolic acids, making any degradation products inherently safe for human consumption. Additionally, even if the entire unrecovered portion migrated into the edible fruit pulp, the resulting concentrations would remain well below the established cytotoxicity threshold of 0.1 mg mL^−1^, which showed no significant adverse effects on the viability of human gastrointestinal epithelial cells. Furthermore, any ingested derivatives would undergo structural decomposition in the gastrointestinal tract under the action of digestive enzymes, further reducing effective exposure levels. These findings collectively demonstrate that the phenolic acid-grafted COS derivative coating at the proposed treatment concentration (2 mg mL^−1^) poses no significant safety risk to consumers, whether the derivatives remain on the fruit surface, degrade into natural small-molecule components, or migrate into the fruit tissue.

2.0 mg mL^−1^ was selected as the optimal concentration for subsequent preservation experiments following comprehensive evaluations of solubility, antibacterial efficacy, and safety. First, this concentration achieved the highest DPPH radical scavenging activity and ferric reducing capacity among the tested concentrations, while also demonstrating superior antibacterial efficacy. Second, preliminary pre-experiments confirmed that concentrations exceeding 2.0 mg mL^−1^ compromised the aqueous solubility of PA-g-COS derivatives, leading to precipitation and uneven distribution on the cherry tomato surface, which would undermine preservation effects. Importantly, safety validation via the CCK-8 assay on GES-1 and NCM-460 cells confirmed the biocompatibility of 2 mg mL^−1^ treatment concentration. This safety profile is consistent with the excellent biocompatibility of phenolic acid-grafted polysaccharide derivatives reported in previous studies,^36^ further supporting the safe application of 2.0 mg mL^−1^ PA-g-COS derivatives in cherry tomato postharvest preservation.

### Quality of cherry tomatoes

#### Decay incidence and browning incidence

**Figure 6:**
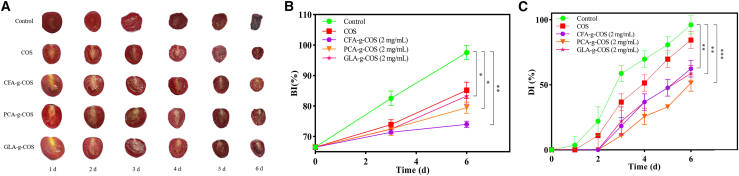
Effects of different PA-g-COS derivatives on the appearance of cherry tomatoes (A) Appearance changes of different PA-g-COS derivatives. (B) Browning incidence (BI) of different PA-g-COS derivatives. (Data are presented as mean ± SD, *n* = 3 independent experiments). (C) Decay incidence (DI) of different PA-g-COS derivatives. (Data are presented as mean ± SD, *n* = 3 independent experiments). *p* value notification: ∗*p* < 0.05, ∗∗*p* < 0.01, and ∗∗∗*p* < 0.001.

The decay incidence (DI) and browning incidence (BI) are primary quality parameters influencing the selection and acceptance of fruits.^37^^,^^38^^,^^39^ As shown in Figure 6, the DI of the control and COS groups reached 22.2% and 11.1% on the second day of storage, respectively, whereas all PA-g-COS groups maintained a DI of 0.0%. By the end of storage, the DI values were 96.0% in the control group, 84.3% in the COS group, 62.3% in the CFA-g-COS group, 51.3% in the PCA-g-COS group, and 58.6% in the GLA-g-COS group. For BI, the corresponding values at the end of storage were 97.5% in the control group, 85.2% in the COS group, 73.9% in the CFA-g-COS group, 79.4% in the PCA-g-COS group, and 83.2% in the GLA-g-COS group. This shows that spraying 2 mg L^−1^ derivatives on cherry tomatoes significantly reduced decay (33.7%–44.7%), browning (14.3%–23.6%).

Previous studies have demonstrated that the postharvest quality of fruits and vegetables is closely associated with transpiration and respiration.^40^^,^^41^ In this study, the DI and BI of cherry tomato increased gradually during storage, but these values were significantly lower in the PA-g-COS groups than in the control and COS groups (*p* < 0.05). This suggests that PA-g-COS derivatives may form a protective film on the fruit surface, isolating the fruit from air to reduce transpiration and respiration rates.^4^ Fruit respiration generates peroxides that induce oxidative damage to cell membranes, promoting the release of phenolic compounds. These compounds react with polyphenol oxidase (PPO), leading to nutritional loss and browning of the fruit’s flesh and peel.^42^ PA-g-COS treatment significantly alleviated fruit decay and browning by inhibiting microbial infection and regulating enzyme activities,^43^ thereby extending the shelf life of cherry tomatoes.

#### Reducing sugar, soluble solids, and titratable acid

**Figure 7:**
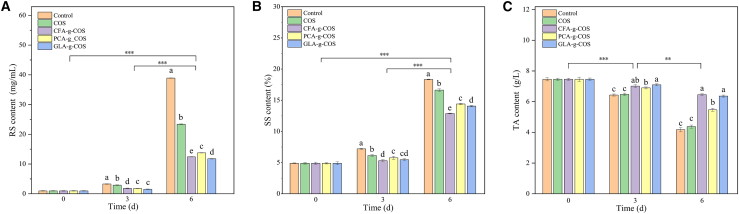
Effects of different PA-g-COS derivatives on the quality of cherry tomatoes (A) Reducing sugars (RSs) of different PA-g-COS derivatives. (Data are presented as mean ± SD, *n* = 3 independent experiments). (B) Soluble solids (SSs) of different PA-g-COS derivatives. (Data are presented as mean ± SD, *n* = 3 independent experiments). (C) Titratable acidity (TA) of different PA-g-COS derivatives. (Data are presented as mean ± SD, *n* = 3 independent experiments). Different lowercase letters indicate significant differences between different treatment groups on the same day (*p* < 0.05). *p* value notification: ∗*p* < 0.05, ∗∗*p* < 0.01, and ∗∗∗*p* < 0.001.

A progressive increase in reducing sugar (RS) content was observed across all five groups during storage, and this upward trend was mitigated in the PA-g-COS treatment groups (Figure 7A). By the 6th day, the RS levels reached 38.9 mg mL^−1^ in the control group, 23.4 mg mL^−1^ in the ungrafted COS group, 12.4 mg mL^−1^ in the CFA-g-COS group, 13.8 mg mL^−1^ in the PCA-g-COS group, and 11.8 mg mL^−1^ in the GLA-g-COS group. Compared to the ungrafted COS group, the PA-g-COS groups exhibited a 41.9%–49.6% reduction in the RS content, indicating the most significant inhibitory effect on RS accumulation. The cumulative increase in RS across all groups is consistent with the respiratory metabolism of postharvest fruits.^44^

Soluble solids (SSs), as key respiratory substrates, reflect fruit maturity and directly influence flavor.^45^ As shown in Figure 7B, the SS content increased gradually across all groups during storage, which is consistent with the findings of Mai A et al.^46^ However, the accumulation rate was slower in phenolic acid-grafted COS groups than in the control and ungrafted COS groups. By the 6th day of storage, the SS contents in the PA-g-COS groups were 21.3%–29.5% lower than those in the control group and 13.3%–22.3% lower than those in the ungrafted COS group. These results demonstrate that PA-g-COS derivatives effectively delayed SS accumulation in postharvest cherry tomatoes. Previous studies have confirmed that reducing sugars account for approximately 55%–65% of the total soluble solids in tomato fruits, supporting a positive correlation between RS and SS changes.^47^ The observed increases in RS and SS during storage are primarily attributed to the hydrolysis of storage polysaccharides (e.g., hemicellulose, pectin, and starch) into oligosaccharides and simple sugars (including RS) in postharvest tomato fruits.^48^ In the present study, PA-g-COS treatments were found to inhibit *α*-amylase activity during postharvest ripening (Figure S2), further confirming its regulatory role in fruit carbohydrate metabolism. PA-g-COS treatments may bind to the active site of α-amylase through multiple interactions, including hydrogen bonds, ionic interactions, π-π stacking, and salt bridges, and interact with the enzyme’s amino acid residues to alter its structure.^49^^,^^50^^,^^51^ This structural modification suppresses polysaccharide hydrolysis and explains the reduced accumulation of RS and SS in PA-g-COS-treated groups.

The titratable acid (TA) content decreased in all groups (Figure 7C), but remained significantly higher in PA-g-COS groups than in the ungrafted COS group throughout the storage period. By the end of the experiment, the TA content in the PA-g-COS groups was 24.9%–47.3% higher than that in the ungrafted COS group. As an important respiratory substrate, TA is gradually consumed during storage to support fruit physiological metabolism.^52^ PA-g-COS treatments significantly mitigated this decline, which is consistent with previous findings.^43^This effect may be attributed to the formation of a surface permeation barrier on cherry tomatoes by PA-g-COS derivatives, which regulates respiratory metabolism to reduce the consumption of organic acids or their conversion to sugars, thereby delaying fruit ripening.

### Microbial indicator of cherry tomatoes

**Figure 8:**
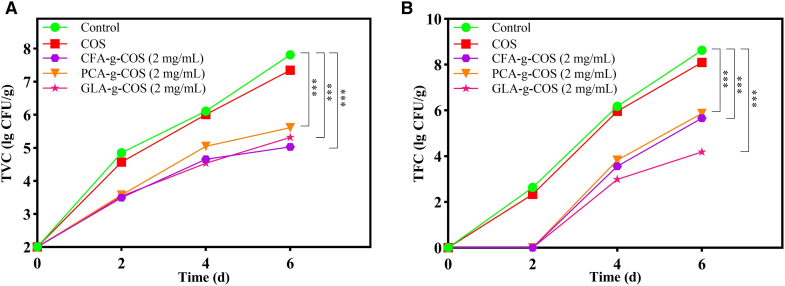
Changes in microbial indicators of cherry tomatoes treated with different PA-g-COS derivatives after 6 days of storage (A) Total viable count (TVC). (Data are presented as mean ± SD, *n* = 3 independent experiments). (B) Total fungal count (TFC). (Data are presented as mean ± SD, *n* = 3 independent experiments). *p* value notification: ∗*p* < 0.05, ∗∗*p* < 0.01 and ∗∗∗*p* < 0.001.

The results of the total bacterial count are presented in Figure 8A. Both the control and COS groups exhibited rapid microbial growth, reaching 7.8 ± 0.009 and 7.3 ± 0.002 lg CFU·g^−1^ on day 6, respectively. Notably, all PA-g-COS derivative groups demonstrated pronounced inhibitory effects on microbial growth. On day 6, the total viable count (TVC) of the CFA-g-COS, PCA-g-COS, and GLA-g-COS groups was 5.0 ± 0.01, 5.6 ± 0.04, and 5.2 ± 0.06 lg CFU·g^−1^, respectively. Among these, the CFA-g-COS group showed the strongest inhibitory activity, with a final TVC ∼2.8 lg CFU·g^−1^ lower than that of the control group (*p* < 0.05). Figure 8B displays the total fungal colony count observed during the storage of cherry tomatoes. All samples showed a significant increase in fungal count as the storage time increased. Nonetheless, the application of PA-g-COS derivatives markedly retarded the increase (*p* < 0.05). At the end of storage, the fungal counts in the control, COS, CFA-g-COS, PCA-g-COS, and GLA-g-COS groups were 8.6 ± 0.008, 8.1 ± 0.02, 5.7 ± 0.02, 5.9 ± 0.01, and 4.4 ± 0.05 lg CFU·g^−1^, respectively. These results indicate that PA-g-COS derivatives exerted a pronounced inhibitory effect on the growth of fungi on tomato surfaces, primarily attributable to their inherent antimicrobial activity.^17^

### Antioxidant of cherry tomatoes

**Figure 9:**
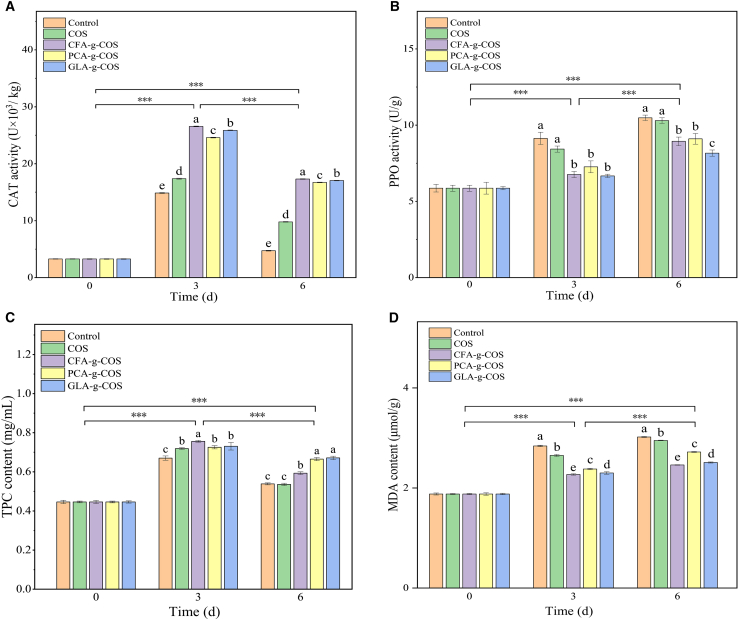
Effects of different PA-g-COS derivatives on the antioxidant activity of cherry tomatoes (A) Catalase (CAT) of different PA-g-COS derivatives. (Data are presented as mean ± SD, *n* = 3 independent experiments). (B) Polyphenol oxidase (PPO) of different PA-g-COS derivatives. (Data are presented as mean ± SD, *n* = 3 independent experiments). (C) Total phenol compounds (TPCs) of different PA-g-COS derivatives. (Data are presented as mean ± SD, *n* = 3 independent experiments). (D) Malondialdehyde (MDA) of different PA-g-COS derivatives. (Data are presented as mean ± SD, *n* = 3 independent experiments). Different lowercase letters indicate significant differences between different treatment groups on the same day (*p* < 0.05). *p* value notification: ∗*p* < 0.05, ∗∗*p* < 0.01, and ∗∗∗*p* < 0.001.

Catalase (CAT), a key antioxidant enzyme, catalyzes the decomposition of hydrogen peroxide (H_2_O_2_) to reduce reactive oxygen species (ROS) accumulation and protecting cells from oxidative damage.^53^ Throughout the entire storage period, CAT activity in all groups showed a trend of initial increase followed by a decline, while PA-g-COS treatments significantly enhanced CAT activity in cherry tomatoes (*p* < 0.05). On the 3rd day of storage, PA-g-COS groups showed 41.5%–52.9% higher CAT activities than the ungrafted COS group. By the end of storage, the PCA-g-COS, GLA-g-COS, and CFA-g-COS groups exhibited 71.0%, 74.2%, and 77.0% higher CAT activity than the ungrafted COS group, respectively (Figure 9A). COS acts as an inducer of plant defense responses, activating the antioxidant system and triggering the rapid production of H_2_O_2_. As a signaling molecule, H_2_O_2_ initiates the ROS scavenging system, thereby upregulating CAT activity. Compared with the ungrafted COS group, the PA-g-COS groups exhibited higher CAT activity, which is likely attributed to the strong antioxidant properties of phenolic acid-grafted COS derivatives.

Polyphenol oxidase (PPO), a key endogenous enzyme, mediates enzymatic browning in fruits and vegetables. Under aerobic conditions, it catalyzes the hydroxylation of monophenols to *o*-diphenols, which are subsequently oxidized to o-quinones. These quinones undergo non-enzymatic polymerization with other quinones, amino acids, and proteins to form brown compounds, resulting in surface browning.^54^ As shown in Figure 9B, PA-g-COS treatments significantly inhibited the elevation of PPO activity. After 6 days of storage, the PPO activity in the CFA-g-COS, PCA-g-COS, and GLA-g-COS groups was 11.7%–20.7% lower than that in the ungrafted COS group (*p* < 0.05), with there was no significant difference between the ungrafted COS and control groups. Phenolic acids (e.g., gallic, caffeic, and cinnamic acid) inhibit PPO by competitively binding its active site (via structural similarity to substrates) or chelating binuclear Cu^2+^, thereby preventing substrates from entering the catalytic center of the enzyme.^55^ Additionally, COS itself also inhibits PPO activity.^51^^,^^56^ Therefore, PA-g-COS treatments effectively reduce PPO activity by minimizing the enzyme’s contact with phenolic substrates, slowing the oxidative degradation of phenolics and ultimately alleviating enzymatic browning.

Phenolic compounds, important plant secondary metabolites, play a crucial role in maintaining fruit quality.^43^ These compounds exhibit significant antioxidant activity, effectively protecting cellular components from oxidative damage.^57^ As depicted in Figure 9C, the total phenolic content (TPC) in all groups exhibited a trend of initial increase followed by a decrease over the storage period. On the 3rd day, the CFA-g-COS group showed the highest TPC (0.76 mg mL^−1^), which was significantly higher than that in the control (0.67 mg mL^−1^) and ungrafted COS groups (0.72 mg mL^−1^) (*p* < 0.05). By the 6th day, the TPC in the CFA-g-COS, PCA-g-COS, and GLA-g-COS groups was 10.6%, 24.1%, and 25.1% higher than that in the ungrafted COS group, respectively. These results indicate that PA-g-COS treatments slowed the degradation of phenolic compounds in cherry tomatoes. Phenylalanine ammonia-lyase (PAL), a key rate-limiting enzyme in the phenylpropanoid pathway, mediates phenolic biosynthesis.^58^ In fresh-cut fruits and vegetables, mechanical damage increases PAL activity to promote phenolic accumulation.^59^ Additionally, PA-g-COS treatments effectively inhibit PPO activity, which slows the oxidative degradation of phenolic^60^ and enhances fruit preservation.

Malondialdehyde (MDA), a hallmark product of membrane lipid peroxidation, directly reflects the extent of oxidative damage to cell membranes through changes in its content changes.^61^ As shown in Figure 9D, the MDA content increased in all groups during storage. The control and ungrafted COS groups exhibited increases of 60.6% and 56.9%, respectively, indicating severe damage to the cell membrane system. However, PA-g-COS groups had 7.8%–16.6% lower MDA content than the ungrafted COS group (2.95 μmol g^−1^) (*p* < 0.05). These findings demonstrated that PA-g-COS treatments effectively inhibit MDA accumulation in cherry tomatoes, thereby alleviating cell damage induced by membrane lipid peroxidation.

### Principal components analysis

**Figure 10:**
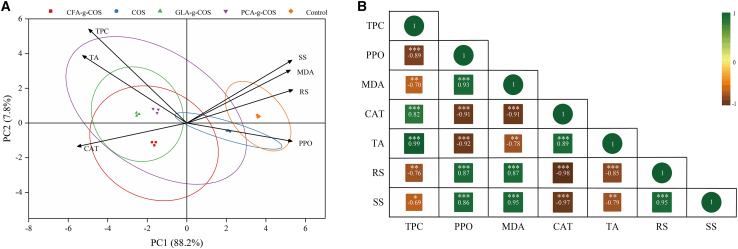
PCA and Pearson’s correlation analysis of cherry tomatoes (A) PCA biplot of cherry tomatoes index parameters for different PA-g-COS derivatives. (B) Heatmap of the Pearson’s correlation analysis of cherry tomatoes index parameters for different PA-g-COS derivatives. *p* value notification: ∗*p* < 0.05, ∗∗*p* < 0.01, and ∗∗∗*p* < 0.001.

To comprehensively understand the complex interactions among these multidimensional indicators and minimize the impact of subjective weighting,^62^ principal component analysis (PCA) was employed in this study. The PCA biplot (Figure 10A) explicitly visualizes the distribution of all treatment groups and the loading vectors of variables across the first two principal components. The first two principal components (PC1 = 88.2%, PC2 = 7.8%) accounted for 96.0% of the total variance in the data. This visualization is critical for interpreting variable contributions, as it reveals a distinct separation of treatment groups based on their antioxidant activity and quality attributes. Treatments with negative scores on PC1 (CFA-g-COS, PCA-g-COS, and GLA-g-COS) clustered closely with variables associated with enhanced antioxidant capacity, including TPC and CAT activity. All of these variables exhibited strong negative loadings on PC1. In contrast, the control and ungrafted COS groups (with positive PC1 scores) aligned with variables linked to MDA and PPO activity, both of which showed positive loadings on PC1. This spatial congruence implies that PA-g-COS application is associated with enhanced antioxidant capacity, resulting in pronounced improvements in the antioxidant performance of cherry tomatoes by increasing beneficial indices (e.g., TPC and CAT) while reducing detrimental ones (e.g., MDA and PPO).

To further elucidate the effects of PA-g-COS treatments on the quality of cherry tomatoes, Pearson correlation analysis was conducted (Figure 10B). TPC showed a significant negative correlation with PPO (*r* = −0.89), MDA (*r* = −0.70), RS (*r* = −0.76), and SC (*r* = −0.69). This suggests that phenolic compounds inhibit oxidative processes, reduce membrane lipid peroxidation, and delay fruit senescence.^63^ TPC was positively correlated with CAT (*r* = 0.82), indicating that higher TPC may be associated with enhanced CAT activity.^24^^,^^64^ CAT was negatively correlated with SS (*r* = −0.97) and RS (*r* = −0.98), while showing a significant positive correlation with TA (*r* = 0.89). These results confirm that the stability of quality can be maintained by enhancing the activity of antioxidant enzymes. These results indicate that maintaining quality stability correlates with enhanced antioxidant enzyme activity, further supporting the preservative efficacy of PA-g-COS treatments.

This study successfully synthesized three phenolic acid-grafted COS derivatives via free radical grafting. FTIR and ^1^H NMR confirmed the successful grafting of the three phenolic acids onto the COS backbone. Post-grafting, the derivatives exhibited significantly enhanced antioxidant and antibacterial activities compared to ungrafted COS. Spraying cherry tomato with 2 mg L^−1^ PA-g-COS derivative solutions effectively reduced decay rate and browning incidence, exerting substantial benefits for postharvest preservation and quality maintenance. The derivatives also maintained titratable acidity, reduced the accumulation of soluble solids and reducing sugars, and delayed fruit ripening and senescence. PA-g-COS treatments alleviated oxidative stress in cherry tomatoes by enhancing catalase activity to inhibit membrane lipid peroxidation, while suppressing polyphenol oxidase activity to slow phenolic degradation. Therefore, phenolic acid-grafted COS derivatives represent an effective strategy to improve the postharvest quality and storage performance of cherry tomatoes, holding promising potential for practical application in fruit preservation.

### Limitations of the study

Phenolic acid-grafted COS derivatives are derived from natural sources, featuring biodegradability and edibility. Nevertheless, systematic safety evaluation remains indispensable when applied in food preservation, even for natural-derived materials, rigorous toxicity assessments are critical to ensure compatibility with human health. As a synthetic preservative, the toxicity of COS-g-phenolic acid to humans should receive further attention from researchers. Chandika et al.^65^ found that the COS-g-sinapic acid showed good cytocompatibility against human dermal fibroblast (HDF) and HaCaT cells. Ngo et al.^66^ found that COS-g-cytotoxicity of gallic acid was non-toxic and biocompatible. Consistent with these findings, our study confirmed that the grafted derivatives exerted no toxic effects on the GSE-1 cells and NCM-460 cells (Figure 5). However, Current research on the toxicity of PA-g-COS has primarily focused on *in vitro* assessments. In contrast, *in vivo* acute toxicity assessment is recognized as the most direct and effective method for biosafety validation. Therefore, future research should focus on conducting further comprehensive evaluations of the biosafety of these derivatives, so as to support their extensive application in the food industry.

## Resource availability

### Lead contact

Further information and requests for resources and reagents should be directed to and will be fulfilled by the lead contact, Nan Wang (wangnan198110@163.com).

### Materials availability

This study did not generate new unique reagents.

### Data and code availability

• Data: The data reported in this article are available within the main text or supplemental information and will be shared by the lead contact upon request.
• Code: This article does not report original code.
• Other items: Any additional information required to reanalyze the data reported in this article will be shared by the lead contact upon request.

## Acknowledgments

Research Foundation of Shaoxing 330 Overseas Talents Program.

## Author contributions

Data curation: R.Z.; funding acquisition, N.W.; investigation: R.L. and S. L.; methodology: R. Z and H. C.; project administration: N.W.; writing – original draft: R. Z. and H. C.; writing – review and editing: N.W. All authors have read and agreed to the published version of the article.

## Declaration of interests

The authors declare no competing interests.

## STAR★Methods

### Key resources table

**Table:** 

REAGENT or RESOURCE	SOURCE	IDENTIFIER
**Bacterial and virus strains**		

*Escherichia coli*	Merck	CAT#EC11303
*Staphylococcus aureus*	Thermo Fisher	CAT#R4607010

**Chemicals, peptides, and recombinant proteins**		

Chitosan Oligosaccharide	Macklin Biochemical Ltd	Cat#523682, CAS: 148411-57-8
Protocatechuic Acid	Macklin Biochemical Ltd	Cat#03930590, CAS: 99-50-3
Gallic Acid	Macklin Biochemical Ltd	Cat# 8.42649, CAS: 149-91-7
Caffeic Acid	Macklin Biochemical Ltd	Cat# 205546, CAS: 331-39-5
2-Thiobarbituric acid	Macklin Biochemical Ltd	Cat#T5500, CAS: 504-17-6
FeSO_4_·7H_2_O	Macklin Biochemical Ltd	Cat#215422, CAS: 7782-63-0
2,4,6-Tris (2-pyridyl)-S-Triazine	Macklin Biochemical Ltd	Cat#T1253, CAS: 3682-35-7
Na_2_CO_3_	Macklin Biochemical Ltd	Cat#1613757, CAS: 497-19-8
Anhydrous Glucose	Macklin Biochemical Ltd	Cat#40139, CAS: 50-99-7
Absolute Ethanol	Macklin Biochemical Ltd	Cat#1.07017, CAS: 64-17-5
Trichloroacetic Acid	Macklin Biochemical Ltd	Cat#6.22342, CAS: 76-03-9
DL-Dithiothreitol	Macklin Biochemical Ltd	Cat#D9779, CAS: 27565-41-9
Polyvinylpyrrolidone	Macklin Biochemical Ltd	Cat#81440, CAS: 25249-54-1
Na_2_HPO_4_·2H_2_O	Macklin Biochemical Ltd	Cat#71643, CAS: 10028-24-7
NaH_2_PO_4_	Macklin Biochemical Ltd	Cat#S3139, CAS: 7558-80-7
NaOH	Macklin Biochemical Ltd	Cat#1.60309, CAS: 1310-73-2
H_3_PO_4_	Macklin Biochemical Ltd	Cat#345245, CAS: 7664-38-2

**Experimental models: Cell lines**		

GES-1	This paper	–
NCM-460	This paper	–

**Software and algorithms**		

IBM SPSS Statistics 27	IBM	https://www.ibm.com/
Origin 2021	OriginLab Corporation	https://www.originlab.com
R version 4.3.1	R Core Team	https://www.r-project.org/

### Experimental model and study participant details

#### Cell lines

Human gastric mucosal epithelial cells (GES-1) and normal human colonic epithelial cells (NCM-460) were obtained from the Engineering Research Center of Industrial Microbiology (Henan Province, China). Both cell lines were routinely maintained in high-glucose Dulbecco’s Modified Eagle’s Medium (DMEM) supplemented with 10% (v/v) fetal bovine serum under standard culture conditions (37°C, 5% CO_2_, humidified atmosphere). Culture medium was refreshed every 48-72 h, and cells were passaged upon reaching 70-80% confluence.

### Method details

#### Preparation of phenolic acid-chitooligosaccharide (PA-g-COS) derivatives

0.5 g of COS was dissolved in 25 mL of distilled water, followed by the addition of 1 ml of 1 mol·L^-1^ hydrogen peroxide solution containing 54 mg ascorbic acid. The mixture was reacted at 25°C for 30 min, and its pH was then adjusted to 5.0. Subsequently, 0.25 g of phenolic acid was added, and the reaction was continuously stirred for 6 h. After the reaction, the mixture was allowed to stand for 30 min and then dialyzed using a 1.5 kDa membrane. The dialyzed solution was freeze-dried to obtain the final products: various phenolic acid-grafted chitooligosaccharide (PA-g-COS), including protocatechuic acid-grafted-COS (PCA-g-COS), caffeic acid-grafted-COS (CFA-g-COS), and gallic acid-grafted-COS (GLA-g-COS).

#### Fourier transform infrared (FTIR) spectroscopy

The functional groups of COS and its derivatives were identified via analysis of characteristic bands in their infrared spectrum. Infrared spectra of the samples were measured using a FTIR spectroscopy (Thermo Fisher Scientific Inc., Massachusetts, USA). For FTIR analysis, pellets were prepared by grinding 1 mg of freeze-dried sample powder with 100 mg of dry potassium bromide (KBr) powder, followed by pressing the mixture in a mold. The spectral acquisition parameters were set as follows: spectral range of 4000-400 cm^-1^, 16 scans, and a resolution of 4 cm^-1^.

#### ^1^H nuclear magnetic resonance (^1^H NMR) spectroscopy

^1^H NMR spectroscopy was employed to characterize the structures of COS and its derivatives.^67^ Freeze-dried samples (10 mg) were dissolved in 50 μL of D_2_O and ^1^H NMR spectra were recorded using an AVANCE III 600 MHz spectrometer (Bruker, Germany) at 25°C.

#### Yields and degree of substitution (DS) analysis

To evaluate the reaction efficiency, the production yield and DS were calculated. The yield is defined as the percent ratio of the actual weight (g) of the final product to its theoretical weight (g). DS was calculated using the following Equation 1:

(Equation 1) $$\text{DS}\;\left(\%\right)=\frac{H_{s}}{H_{1}}\times 100$$

where *H*_*s*_ represents the integral area of the hydrogen atom in the benzene ring of the derivatives, *H*_*1*_ represents the integral area of the 1-position carbon-bonded hydrogen proton on the main chain of the COS.^17^

#### Antioxidant activity determination

The 1,1-Diphenyl-2-picrylhydrazyl (DPPH) radical scavenging activity was determined using the method described by Sun et al.^68^ with minor modification. Samples were lyophilized to constant weight and initially prepared as a 10 mg·mL^-1^ stock solution. This stock solution was then serially diluted with deionized water to obtain working solutions of different concentrations (0.8, 1.2, 1.6 and 2.0 mg·mL^-1^). For the assay, 1 mL of each working solution was mixed with 2.0 mL of 0.1 mM DPPH ethanol solution. For the control group, the DPPH solution was replaced with 2.0 mL of anhydrous ethanol; for the blank group, the sample solution was replaced with 1.0 mL deionized water. All mixtures were incubated in the dark at 25°C for 30 minutes, after which the absorbance was measured at 517 nm. The DPPH radical scavenging activity was calculated using the following Equation 2 and expressed as a percentage (%):

(Equation 2) $$S\text{cavenging}\;\text{effect}\;\left(\%\right)=\left[1-\frac{A_{control}-A_{sample}}{A_{blank}}\right]\times 100$$

where *A*_*control*_ was the absorbance of the control group, *A*_*sample*_ was the absorbance of the sample group, and *A*_*blank*_ was the absorbance of the blank group.

The ferric reducing antioxidant power (FRAP) assay was determined using the method of Xu et al.^69^ with minor modifications. The FRAP working solution was prepared by mixing 0.3 mol·L^-1^ acetate buffer (pH 3.6), 10 mmol·L^-1^ 2,4,6-Tripyridyl-s-Triazine (TPTZ), and 20 mmol·L^-1^ FeCl_3_·6H_2_O at a volume ratio of 10:1:1. This mixture was incubated in a water bath at 37°C for 10 minutes. Subsequently, 3 mL of the preheated working solution was mixed with 1 mL of the sample solution under nitrogen protection, followed by a 30 minutes water bath reaction at 37°C. The absorbance was measured at 593 nm using a TU-1901 spectrophotometer (Puxi, China) and the results were expressed as mmol·L^-1^.

#### Determination of minimum inhibitory concentration (MIC) and zone of inhibition

Gram-negative *Escherichia coli* (*E. coli*) and Gram-positive *Staphylococcus aureus* (*S. aureus*) were selected as target bacteria to evaluate antibacterial activity of phenolic acid-g-COS via the minimal inhibitory concentration (MIC) and inhibitory zones. The MIC was defined as the lowest concentration of the derivatives at which bacterial growth was not visible to the naked eye. Briefly, the samples were dissolved in water at a concentration of 0.8, 1.2, 1.6, and 2.0 mg·mL^-1^, then 15 mL of nutrient agar before solidifying was mixed well with 1 mL of sample solution and set the mixture to solidify at room temperature. Bacteria culture (0.1 mL, 10^6^ -10^7^ CFU·mL^-1^) grown in Petri dish was incubated at 37°C for 24 h. For inhibitory zones, the nutrient agar medium in Petri dish was inoculated with 0.1 mL of 10^7^-10^8^ CFU·mL^-1^ bacteria. Three sheets of filter paper with a diameter of 6 mm were immersed in sample solution (20 mg·mL^-1^) for 30 min and then placed on microbial cultures. Bacteria strains were incubated at 37°C for 24 h. The diameter of the zone of inhibition was measured using a caliper.

#### Biocompatibility determination

Human gastric mucosal epithelial cells (GES-1) and normal human colonic epithelial cells (NCM-460) were acquired from the Engineering Research Center of Industrial Microbiology (Henan Province, China). For cytotoxicity evaluation, GES-1 and NCM-460 cells were seeded in 96-well plates at a density of 9.0×10^3^ cells per well, followed by a 24 h of incubation to ensure full cell adherence. After the establishment of blank and vehicle control groups, cells were treated with test compound solutions at concentrations of 1.0, 0.5, 0.25, 0.1, and 0.05 mg·mL^-1^ for 24 h. Subsequently, cell viability was measured using the Cell Counting Kit-8 (CCK-8) assay according to the manufacturer’s protocol. All treatments were performed in triplicate to characterize dose-dependent effects on cell proliferation.

#### Fruit preservation analysis

Cherry tomatoes were collected from Zhongwang Ecological Far in Jiangbei District, Ningbo City, Zhejiang Province. After harvesting, cherry tomatoes were first sorted to remove fruits with mechanical damage, disease spots, or uneven maturity, retaining those with uniform shape, size, and color. They were then gently rinsed with distilled water to remove surface impurities and air-dried at room temperature for 30 min to eliminate surface moisture. The COS and PA-g-COS treatment solutions were applied by spraying using an upper pot spray gun (W71, 0.29 kW; Anasite Iwata Coating Machinery Co., Ltd, China) to achieve uniform coverage of the fruit surface, with a spraying distance of ∼20 cm. After spraying, the fruits were stored at room temperature (23°C and 85% RH) in the dark. Surface decay was visually monitored daily, and the fruits were photographed to document their morphological changes. Cherry tomatoes sprayed with distilled water were designated as the blank group, while those sprayed with COS solution alone served as the positive control group. The overall experimental flow chart is shown in Figure S3.

#### Measurement of decay incidence (DI) and browning incidence (BI)

The decay of cherry tomatoes was evaluated by determining the decay incidence (DI), which was expressed as a percentage (%) following the methodology provided by Shakir et al.,^70^ utilizing Equation 3:

(Equation 3) $$\text{DI}\;\left(\%\right)=\frac{n}{N}\times 100$$

where *n* was the number of rotten fruits, *N* was the initial number of fruits in each group.

The browning incidence (BI) was calculated based on the L∗, a∗, and b∗ values of cherry tomatoes measured with a colorimeter (CR 400 Konica Minolta, Japan), using Equation 4:

(Equation 4) $$\text{BI}\;\left(\%\right)=\frac{X-0.31}{0.172}\times 100$$

where X = (a∗ +1.75L∗)/(5.645L∗ + a∗ - 3.02b∗).

#### Measurement of soluble solids (SS), titratable acidity (TA), and reducing sugar (RS)

The soluble solids (SS) was measured using a PAL-3 refractometer (Atago, Japan) at 25°C.

The titratable acidity (TA) was determined by titration: 1 mL of cherry tomato juice was diluted with 50 mL of deionized water, and titrated with 0.1 mol·L^-1^ NaOH using 1% phenolphthalein as an indicator.^71^ The acidity was expressed as mg of citric acid per 100 g fresh weight (FW).

The reducing sugar (RS) content was determined by the 3,5-dinitrosalicylic acid (DNS) method^72^ with slight modifications. 1 mL of soluble sugar extract filtrate and 1.5 mL of DNS reagent were transferred to test tubes, which was then heated in boiling water bath for 15 min to initiate the reaction. After cooling to room, the absorbance of the sample was measured at 540 nm using a UV-Vis spectrophotometer (TU-1901, Puxi, China). The results were expressed as grams of glucose per 100 g FW.

#### Measurement of microbial indicator

5 g of cherry tomato sample was macerated in 45 mL of 0.89% sterile saline and homogenized for 5 min using aseptic homogenizer. 0.1 mL of the diluted bacterial suspension was inoculated onto plate count agar (PCA) plates, and the total number of bacterial colonies was counted after 48 h of incubation at 30°C. The count of mold and yeast was determined by pouring the diluted sample onto potato dextrose agar plates containing 0.1 g L^-1^ chloramphenicol, and the colonies were counted after 5 days of incubation at 25°C.

#### Measurement of total phenol compounds (TPC) and malondialdehyde (MDA)

The total phenolic content (TPC) was determined using the Folin-Ciocalteu method with slight modifications.^73^ Briefly, 1 g of cherry tomato flesh was homogenized with 5 mL of distilled water, incubated in an 80°C water bath for 40 min, and then centrifuged at 5000 rpm for 5 min. The reaction mixture was prepared by mixing 1 mL of the supernatant, 1 mL of Folin-Ciocalteu reagent, and 4 mL of 10% Na_2_CO_3_ (w/v) solution at a volume ratio of 1:1:4, followed by incubation at room temperature for 30 min. The absorbance was measured at 760 nm, and TPC was calculated using a gallic acid standard curve, expressed as mg gallic acid equivalents per kg FW.

The malondialdehyde (MDA) content was measured using the method described by Jahani et al.^52^ For sample preparation, 1 g of cherry tomato flesh was homogenized with 5 mL of 100 g·L^-1^ trichloroacetic acid (TCA) solution and centrifuged at 10000 rpm for 15 minutes at 4°C. A 2 mL of the supernatant was mixed with an equal volume of 6.7 g·L^-1^ TBA solution, heated in a boiling water bath for 20 min, cooled to room temperature, and then centrifuged at 5,000 rpm for 5 min. The absorbance of the reaction mixture was determined at 450 nm, 532 nm, and 600 nm. For the blank control, 2 mL of TCA solution was used instead of the supernatant, with all other steps unchanged. The MDA content was calculated using Equation 5:

(Equation 5) $$\text{MDA}\;\left({\mu \text{mol}\;g}^{-1}\right)=\frac{c\times V}{V_{s}\times m}$$

where *c* = 6.45 × (A_532_ - A_600_) -(0.56×A_450_), A_532_, A_600_ and A_450_ are the absorbance values at 450 nm, 532 nm, and 600 nm; *V* is the total volume of the sample extract solution (mL); *V*_*s*_ is the volume (mL) of the extraction solution absorbed during the assay; and *m* is the mass of the sample (g).

#### Measurement of catalase (CAT) and polyphenol oxidase (PPO)

CAT activity was determined according to Jahani et al.,^52^ with modifications. 5 g of cherry tomato flesh was homogenized with 5 mL of 0.1 mol L^-1^ sodium phosphate buffer (pH 7.5) containing 5 mmol·L^-1^ dithiothreitol and 5% polyvinylpyrrolidone, thoroughly mixed in an ice bath, and centrifuged at 10,000 rpm for 30 minutes. A 0.5 mL of the supernatant was mixed with 2.9 mL of 20 mmol·L^-1^ hydrogen peroxide solution, and absorbance was measured at 240 nm. CAT activity was expressed as U×10^3^·kg^-1^ FW.

PPO was estimated using the method of Derardja et al.^74^ with a slight modification. 5.0 g of cherry tomato flesh was pulverized with 10 mL of 0.1 mol·L^-1^ phosphate buffer (pH 6.8). After centrifuging, the supernatant (0.1 mL) was mixed with 0.1 mol·L^−1^ phosphate buffer (3.9 mL) and 0.01 mol·L^-1^ catechol (1.0 mL), then reacted for 10 min at 25°C. PPO was determined by measuring absorbance at 525 nm and expressed as U·g^-1^ FW.

### Quantification and statistical analysis

Principal Component Analysis (PCA) was employed to identify differences in antioxidant activity, quality attributes, and biochemical parameters across various PA-g-COS derivative treatments (CFA-g-COS, COS, GLA-g-COS, PCA-g-COS, and Control). Raw variables were subjected to Z-score standardization (mean = 0, SD = 1) to eliminate the interference of dimensional differences among variables and ensure equal weighting in the analysis. Subsequently, a covariance matrix was constructed using the standardized data to quantify the linear correlation strength between each pair of variables. Eigenvalue decomposition was performed on the covariance matrix to extract eigenvalues and corresponding eigenvectors—eigenvalues represent the variance explained by each principal component (PC), while eigenvectors define the direction of each PC in the original variable space. Based on the Kaiser-Harris criterion, principal components with eigenvalues greater than 1 were selected for further analysis. The contribution rate of each retained PC was calculated as the ratio of its eigenvalue to the sum of all eigenvalues, and the cumulative contribution rate was used to evaluate the total variance explained by the selected PCs. Pearson correlation analysis calculated the correlation coefficients (*r*) and p-values to quantify the strength and direction (positive or negative) of associations among variables. One-way analysis of variance (ANOVA) was used to analyze differences in antioxidant activity and quality attributes among five treatments (CFA-g-COS, PCA-g-COS, GLA-g-COS, COS and control). Following significant ANOVA results (*P* < 0.05), Tukey’s test was applied to identify specific treatment differences. All experiments were performed at least three times, and the data were presented as mean ± standard deviation of three or more independent experiments. Data analysis was conducted using SPSS (version 27), Origin 2021 and R version 4.3.1 was used for plotting.
