# Supplementary material for: Modification of chitooligosaccharide with phenolic acids for extended cherry tomato preservation and quality retention
Source: iScience. 2026 Jan 24;29(3):114797. doi: 10.1016/j.isci.2026.114797 (PMC12925054; doi:10.1016/j.isci.2026.114797)
Supplement: Document S1. Figures S1–S3 and Table S1 [file mmc1.pdf]

iScience, Volume *29*

Supplemental information

Modification of chitooligosaccharide with phenolic acids for extended cherry tomato preservation and quality retention

Ruyi Zhang, Hui Chen, Ruohui Li, Shuyan Lin, and Nan Wang

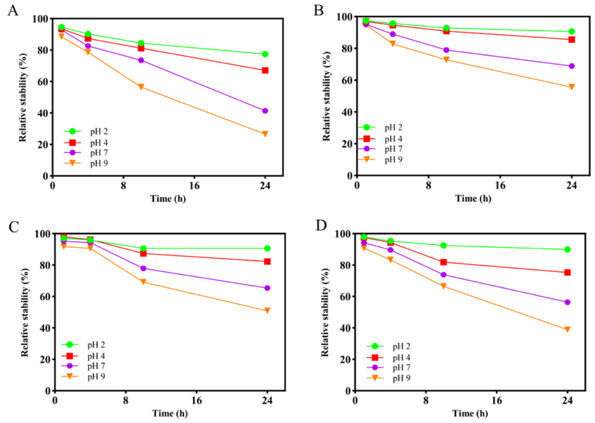

Figure S1. The effect of pH on the stability of different PA-g-COS derivatives

(A) COS.

(B) CFA-COS.

(C) PCA-COS.

(D) GLA-COS.

Data are shown as mean ± standard deviation.

Data are shown for n ≥ 2 independent experiments.

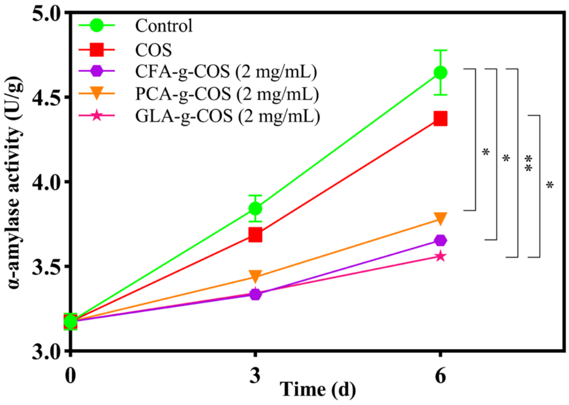

Figure S2. The effect of different PA-g-COS derivatives on the α-amylase

P value notification: *$P < 0.05$, **$P < 0.01$ and ***$P < 0.001$.

Data are shown as mean ± standard deviation.

Data are shown for n ≥ 2 independent experiments.

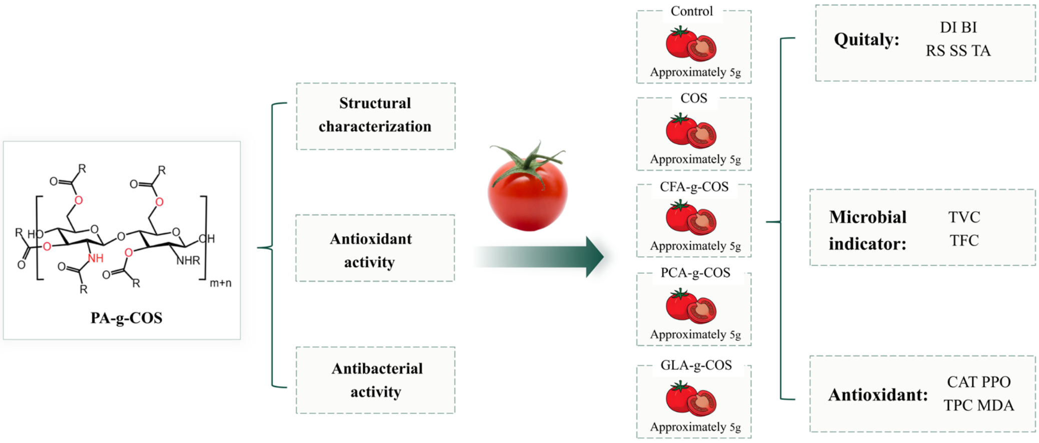

Figure S3. Schematic diagram of the research process

Table S1. Antibacterial activities of different PA-g-COS derivatives

Parameters	Samples	*Escherichia coli*	*Staphylococcus aureus*
MIC (mg/mL)	COS	2	2
	PCA-g-COS	1.6	1.6
	CFA-g-COS	1.2	1.2
	GLA-g-COS	0.8	0.8

Data are shown as mean ± standard deviation.

Data are shown for n ≥ 2 independent experiments.
